# Polymorphisms at activated protein C cleavage sites of factor V: Are they important in the absence of factor V Leiden?

**Published:** 2017-01-05

**Authors:** Ehsan Kheradmand, Shaghayegh Haghjooy-Javanmard, Leila Dehghani, Mohammad Saadatnia

**Affiliations:** 1 Neurosciences Research Center, Alzahra Research Institute, Isfahan University of Medical Sciences, Isfahan, Iran; 2 Applied Physiology Research Center, Isfahan University of Medical Sciences, Isfahan, Iran; 3 Department of Tissue Engineering and Regenerative Medicine, School of Advanced Technologies in Medicine, Shahid Beheshti University of Medical Sciences, Tehran, Iran

**Keywords:** Cerebral Venous Thrombosis, Factor V, Activated Protein C Resistance, Cleavage Sites

## Abstract

**Background:** Activated protein C (APC) inactivates factor V (FV) by cleavage of its heavy chain at Arg306, Arg506, Arg679, and Lys994. Mutational changes, which abolish APC cleavage sites, may predispose thrombosis by altering the inactivation process of FV. FV Leiden (FVL) (Arg506Glu) has been demonstrated as a strong risk factor for thrombosis. In the current study, we have studied whether mutations in the cleavage sites of FV for APC, not due to FVL, would have a role in presenting APC resistance (APCR) and initiation of a cerebral thrombotic event.

**Methods:** A group of 22 patients with a history of cerebral venous thrombosis (CVT), who were not carriers of FVL enrolled in the study. The patients who had conditions associated with acquired APCR were excluded from the study. APCR test was performed on the remaining 16 patients, which showed APCR in 4 plasma samples. DNA sequencing was performed on four exons of FV of APCR patients, encoding Arg306, Arg506, Arg679, and Lys994.

**Results:** Mutations were not found within nucleotides encoding the cleavage sites; neither was found within their close upstream and downstream sequences.

**Conclusion:** Our results show that polymorphisms affecting cleavage sites of FV other than Arg506Glu it would be less likely to be the basis for APCR and its increased thrombosis susceptibility. In addition, it emphasizes on the importance of screening for APCR in the patients diagnosed with CVT.

## Introduction

Cerebral venous thrombosis (CVT) is an uncommon but serious type of cerebral vascular events which mostly affects young adults and children.^1^ It can be caused or predisposed by several medical and surgical disorders such as head trauma, central nervous system infections, malignancies, hematologic and inflammatory disorders. Furthermore, the risk for developing a CVT is higher in patients with hypercoagulable states such as hyperhomocysteinemia, protein C, protein S and antithrombin III deficiencies, as well as careers of prothrombin gene and factor V Leiden (FVL) mutation.^1^^,^^2^ Accordingly, the prothrombotic screening for hypercoagulable states due to genetic and acquired disorders is an important part of investigating the etiology of CVT.^1^^,^^2^

Activated protein C (APC) plays a key role in the anticoagulant pathway by inactivating FVa and Factor VIIIa. There are several proteolytic cleavage sites on FV that is responsible for activation and inactivation of the molecule.^3^ APC can down regulate FV procoagulant activity by cleavage at Arg306, Arg506, Arg679, and Lys994.^3^^,^^4^ Mutational changes which abolish APC cleavage sites may dilute its effect on inactivation of FV. FVL, which is the result of a single point mutation at Arg506, is a common and strong hereditary risk factor for thrombosis in Caucasians.^5^^,^^6^ The APC resistant (APCR) phenotype which is associated with a 7-fold increase in the risk of deep vein thrombosis, is considered to be a consequence of FVL in 95% of cases.^5^

Acquired conditions including cancer, pregnancy, using oral contraceptive pills (OCP) and hormone replacement therapy (HRT), elevated Factor VIII levels ≥ 150 IU/dl and positive lupus anticoagulant results may also be the cause of APCR.^7^^,^^8^

The functional assays that are being used for screening of APCR are widely considered equal to FVL screening test, especially in populations with high prevalence of FVL.^9^ However, DNA-analysis has shown that some cases with APCR do not carry an R506Q mutation.^10^ Therefore, in these individuals, the inactivation of FVa by APC may be influenced by polymorphisms other than Leiden mutation. In the current study, we have studied whether mutations in the cleavage sites of FV for APC, not due to FVL, would have a role in presenting APCR and initiation of a cerebral thrombotic event.

## Materials and Methods

A group of 38 ≤ 65 year-old adults with a past diagnosis of CVT was recalled for this study. These patients had previously been checked for the presence of FVL using restriction fragment length polymorphism (RFLP) method, and the results were negative.^11^ 22 patients accepted to take part in the current study performed at the Neurology Clinic of Alzahra University Hospital (Isfahan, Iran). Of each patient, a 5 cc sample of venous bloodstream was collected into a citrated tube. Poor platelet plasma was extracted after centrifuging and stored at −20 °C. Another 5 cc sample was collected in ethylenediamine tetraacetic acid tube and stored at −20 °C until molecular lab assessments.

Factor VIII, lupus anticoagulant and APCR tests were performed on 22 plasma samples of patients. To eliminate the conditions that could induce APCR without a Leiden mutation, the patients who had ≥ 150 IU/dl of Factor VIII or positive lupus anticoagulant test results were excluded from the study. None of them were pregnant, OCP consumers or under HRT at the time of sampling.

Using HEMOCLOT Factor V-L kit (Aniara, USA), APCR was measured in the plasma samples of the patients. The ratio of clotting time was calculated in the presence and absence of APC. If this ratio was ≥ 2, the plasma is considered normal. In patients with significant APCR such as R506Q mutation, this ratio is lowered to ≤ 1.80. The ratio > 1.80 and < 2 is considered as borderline.

DNA was extracted from blood samples of 4 APCR patients with a standard DNA isolation kit (PrimePrep, Genet Bio, South Korea). Four primers were used for running PCR on the cleavage site regions: 

A 228 bp fragment of exon 7, encoding Arg306 cleavage site, was amplified by the following primer: Forward: 5’-CTTGAACCTTTGCCCAGTGG-3’ and reverse: 5’-TTGTCTTTCTGTCCTAACTCAGC-3’.^12^ A 267 bp fragment of exon 10, encoding Arg506 cleavage site, was amplified by following primer: Forward: 5'-TGCCCAGTGCTTAACAAGACCA-3’ and reverse: 5'-TGTTATCACACTGGTGCTAA-3’.^5^ A 226 bp fragment of exon 13, encoding Arg679 cleavage site, was amplified by the following primer: Forward: 5’-TGTCTGTCTCTCTTCTGTAAGAACT-3’ and reverse: 5’-CTGGTAATCATAGTCAGCATCAC-3’.^12^ The last primer: Forward: 5’-CAGCCCCCAGAATGCCTCA-3’ and reverse: 5’- AGACCTGGAGGACAGCTTGC-3’ was designed to synthesize a 432 bp fragment of exon 13, that encodes Lys994 cleavage site.

Polymerase chain reaction products were collected into sterile 500 µl tubes and stored in −20 °C until sequencing procedures (ABI 3730XL DNA Analyzer, Bioneer, South Korea). The results were compared with sequence information of FV in the National Centre for Biotechnology Information (NCBI) gene database.

## Results

A total of 22 patients with a history of CVT, who were negative for FVL, were studied. There were 16 females (72.7%) and 6 males (27.3%) with a mean age of 36.77 ± 13.35 years. Three patients had a positive lupus anticoagulant test and were excluded from the study. The level of Factor VIII was increased in 4 patients, and they were excluded too (one patient excluded for both). 

Four patients out of remaining 16 patients (25%) showed resistance to APC in the absence of other acquired conditions by showing a clotting time ratio under 2. Two of them represented APCR by 1.4 and 1.6 clotting time ratio. The other two patients were in the borderline range (ratio 1.9).

DNA sequencing was done on four regions of FV that encode Arg306, Arg506, Arg679, and Lys994 cleavage sites in four APCR patients. Mutations were not found within nucleotides encoding the cleavage sites; neither was found within their close upstream and downstream sequences.

## Discussion

In this series of patients, the FV-mediated APCR was investigated in a selected group of CVT patients with no evidence for acquired causes of this thrombogenic condition. The sequencing study of four known cleavage sites of FV for APC, as potential sites for generating APCR, failed to find any polymorphism. Similar to the results of the previous testing with RFLP method, DNA sequencing on Arg506 confirmed the absence of FVL mutation. Thus, the APCR could not be explained by a molecular mechanism affecting the APC cleavage sites other than R506Q.

Mutated FVL which decreases the anticoagulant activity of APC by altering the main cleavage site of APC on FV, has been proposed to be strongly associated with CVT.^13^^,^^14^ It has been reported that 10-25% of CVT patients are carriers of this mutation.^14^^,^^15^ Among Iranian population, Rahimi, et al.^16^ found a significant correlation between FVL mutation and CVT in Iranian patients with Kurdish ethnic background. In contrast a study by Ashjazadeh, et al.^17^ indicated that frequency of FVL mutation were not significantly increased in CVT patients who lived in southern Iran with predominant Fars ethnicity.

Similarly, we observed a frequency of 5% in a group of 40 CVT patients and could not find a significant difference compared with controls.^11^ As APCR was observed in some of our patients in the absence of FVL, looking for other genetic polymorphisms appeared to be the most logical approach. Mostly the polymorphisms that potentially act in a similar way as FVL does.

Our exclusion criteria resulted in a small group of 4 patients to be investigated out of 22 recalled CVT patients without FVL, but simultaneously, it would raise the possibility that APCR was a result of a genetic defect.

The cleavage of FV by APC at the cleavage site Arg506 is the most important part in the inactivation of FVa and results in a partial inactivation. It is also essential for optimal exposure of cleavage sites at Arg306 and Arg679 and subsequent inactivation of FVa.^4^^,^^18^ The latter two cleavage sites are associated with cofactor inactivation in which cleavage at Arg306 is responsible for 70% loss of cofactor activity and the subsequent cleavage at Arg679 that occurs at a slower rate than that of Arg306 and Arg506, is responsible for the remaining 30%.^18^ Although there is a lack of studies on kinetics and clinical significance of APC-mediated cleavage at Lys994 in the literature, it appears to be a location of minor importance on cofactor activity of the molecule.^19^

Although the mutation at position Arg506 leads to a significant APCR and associated with 3- to 7-fold increase in the risk of thrombosis as heterozygous and up to 80 folds as homozygous,^20^ two reported polymorphisms at Arg306 have shown a milder APCR phenotype. Arg306→Thr (FV Cambridge) makes a mild APCR and Arg306→Gly (FV Hong Kong) appeared to have no change in the susceptibility to APC cleavage.^21^ Mutations at Arg306 are probably associated with low thrombotic risk.^21^^,^^22^

Theoretically, the mild APCR observed in our patients can be attributed as a result of other unknown cleavage sites on FV. The recent studies have demonstrated some novel cleavage sites close to Arg306 and Arg506.^23^^,^^24^ Hence, the APC cleavage is occurred very slower in these sites and to our knowledge have no physiologic significance in normal situation.^23^^,^^24^ They may have a role in the presence of mutant factors (Leiden, Cambridge, Hong Kong), but still remains to be studied.

The observed APCR could be attributed to other variants of v that display APCR phenotype without interfering its cleavage sites on FV. FV Liverpool^25^ is a mutated variant that carries aIle359Thr substitution which provides an additional glycosylation at Asn357 that results in impaired inactivation of FVa. In addition, it appears to have a poor APC cofactor activity.^25^^,^^26^ The FV R2 haplotype, which is a collection of mutations in FV, also may represent a mild APCR in homozygous carriers.^27^

## Conclusion

Our study could not find any genetic polymorphism of FV, at its multiple cleavage sites for APC. It shows that polymorphisms affecting cleavage sites of FV other than Arg506Glu it would be less likely to be the basis for APCR and its increased thrombosis susceptibility. In addition, as FVL and APCR are considered as independent risk factors for thrombosis,^28^ our results stress the importance of APCR screening in patients with CVT.

## Notes:

**How to cite this article:** Kheradmand E, Haghjooy-Javanmard S, Dehghani L, Saadatnia M. Polymorphisms at activated protein C cleavage sites of factor V: Are they important in the absence of factor V Leiden? Iran J Neurol 2017; 16(1): 30-3.
